# Supplementary material for: Results of PD-L1 Analysis of Women Treated with Durvalumab in Advanced Endometrial Carcinoma (PHAEDRA)
Source: Cancers (Basel). 2022 Dec 30;15(1):254. doi: 10.3390/cancers15010254 (PMC9818816; doi:10.3390/cancers15010254)
Supplement: Supplementary file 1 [file cancers-15-00254-s001.zip › cancers-2054246-supplementary.pdf]

PD-L1 Immunohistochemistry Staining Protocol.

The slides were baked in a fan forced oven for 20 min @68 degrees. Retrieval and staining were performed on Benchmark ULTRA IHC/ISH autostainer using OptiView kit.

Baking- 60 degrees for 12 minutes

Deparaffinization- EZI prep- 72 degrees

Retrieval- CC1@100 degrees- 56 minutes

Peroxidase Inhibitor- 4 minutes

Antibody- 16 minutes@ 36 degrees

Universal Linker- 8 minutes

HRP Multimer- 8 minutes

On board counterstain-

Haematoxylin- 8 minutes

Bluing- 8 minutes

Table S1: Characteristics of the cohort with PD-L1 data available from the PHAEDRA trial

**Baseline characteristics**	**PD-L1 data available (N=67)**
MMR status	
dMMR	34 (51%)
pMMR	33 (49%)
Age in years	67 [60.0, 71.5]
Body Mass Index (kg/m^2)	26.4 [23.5, 34.4]
Current smoker	17 (25%)
ECOG status	
0	33 (49%)
1	31 (46%)
2	3 (5%)
Histology	
Endometroid	51 (76%)
Serous	11 (16%)
Other	4 (6%)
**Outcomes**	
OTRR	17 (25%)
Progression	52 (78%)
Deaths	37 (55%)

Statistics are given as number (percent) or median (interquartile range).

Table S2: Logistic regression models for response

	Univariate models		Models adjusted for MMR status	
Model	OR (95% CI)	p-value	OR (95% CI)	p-value
dMMR	28.4 (5.2 – 534.7)	0.002	-	-
TC+ ≥ 1	2.00 (0.66 – 6.23)	0.22	-	-
ICP ≥ 10	5.13 (1.57 – 17.58)	0.007	2.89 (0.76 – 11.60)	0.12
IC+ ≥ 35	1.48 (0.40 – 4.99)	0.54	-	-
OC algorithm‡	6.92 (1.71 – 46.90)	0.02	6.25 (1.32 – 46.44)	0.035
UC algorithm†	3.90 (1.26 – 13.1)	0.02	3.25 (0.88 – 13.01)	0.08

TC+: tumor cells with positive staining, ICP: percentage of tumor area occupied by immune cells, IC+: percentage of tumor-associated immune cells with positive staining

† Urothelial cancer (UC) algorithm is: TC+ ≥ 25% or ICP>1& IC+ ≥ 25 or ICP = 1 & IC+ = 100

‡ Optimal cut point (OC) algorithm is TC+ ≥ 1 or ICP ≥ 10 or IC+ ≥ 35

Table S3: Progression free survival (PFS) and Overall Survival (OS) Cox Proportional Hazards models

**PFS**	Univariate models		Models adjusted for MMR status	
	HR (95% CI)	p-value	HR (95% CI)	p-value
pMMR	2.99 (1.61, 5.57)	<0.001		
TC+ ≥ 1	0.72 (0.40, 1.27)	0.3		
ICP ≥ 10	0.41 (0.21, 0.83)	0.013	0.59 (0.28, 1.23)	0.16
IC+ ≥ 35	0.79 (0.41, 1.54)	0.5		
OC algorithm‡	0.60 (0.33, 1.07)	0.084		
UC algorithm†	0.50 (0.29, 0.87)	0.015	0.75 (0.42, 1.36)	0.35
**OS**				
pMMR	2.95 (1.42, 6.11)	0.004		
TC+ ≥ 1	0.62 (0.31, 1.24)	0.18		
ICP ≥ 10	0.46 (0.19, 1.10)	0.075		
IC+ ≥ 35	0.60 (0.26, 1.38)	0.23		
OC algorithm‡	0.42 (0.20, 0.86)	0.019	0.53 (0.25, 1.12)	0.10
UC algorithm†	0.54 (0.28, 1.04)	0.066		

TC+: tumor cells with positive staining, ICP: percentage of tumor area occupied by immune cells, IC+: percentage of tumor-associated immune cells with positive staining

† Urothelial cancer (UC) algorithm is: TC+ ≥ 25% or ICP>1& IC+ ≥ 25 or ICP = 1 & IC+ = 100

‡ Optimal cut point (OC) algorithm is TC+ ≥ 1 or ICP ≥ 10 or IC+ ≥ 35

(A)

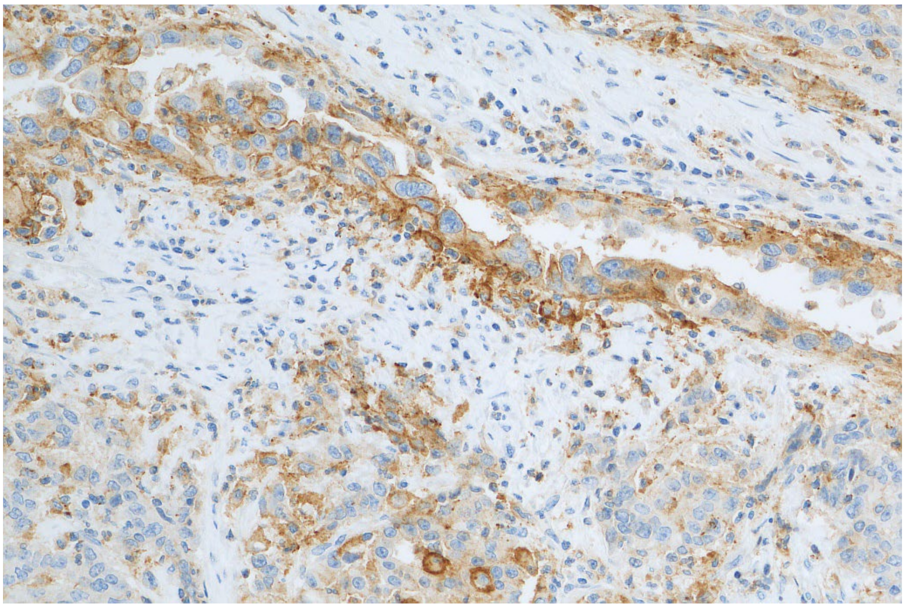

(B)

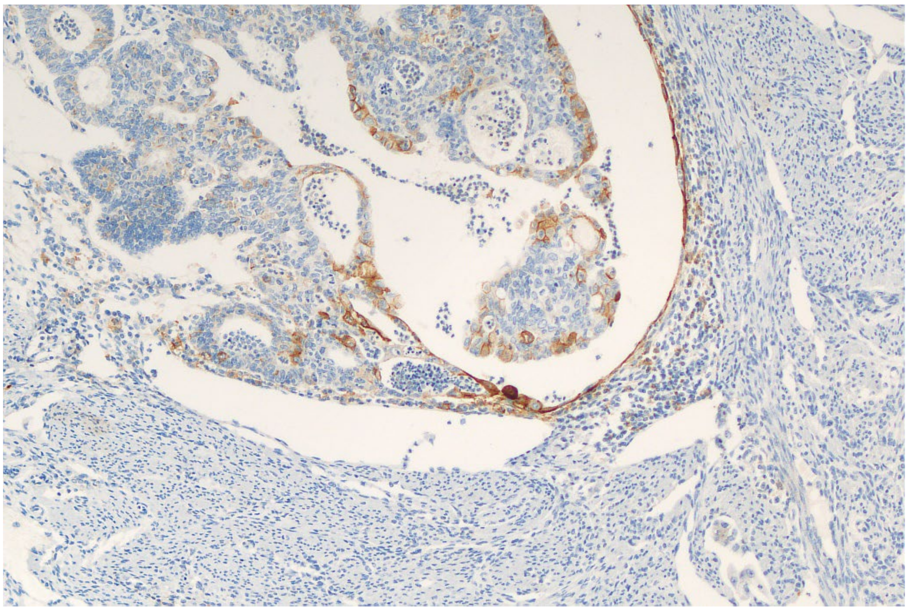

Figure S1: Examples of PD-L1 immunohistochemical staining and individual component scoring. (A) Reviewer 1: TC+ 30, ICP 15, IC+ 30; Reviewer 2: TC+ 40, ICP 25, IC+ 40. (B) Reviewer 1: TC+ 1; ICP 2, IC+ 2; Reviewer 2: TC+1, ICP 5, IC+ 30. (A x200, B x100, C x200). TC+: tumor cells with positive staining, ICP: percentage of tumor area occupied by immune cells, IC+: percentage of tumor-associated immune cells with positive staining

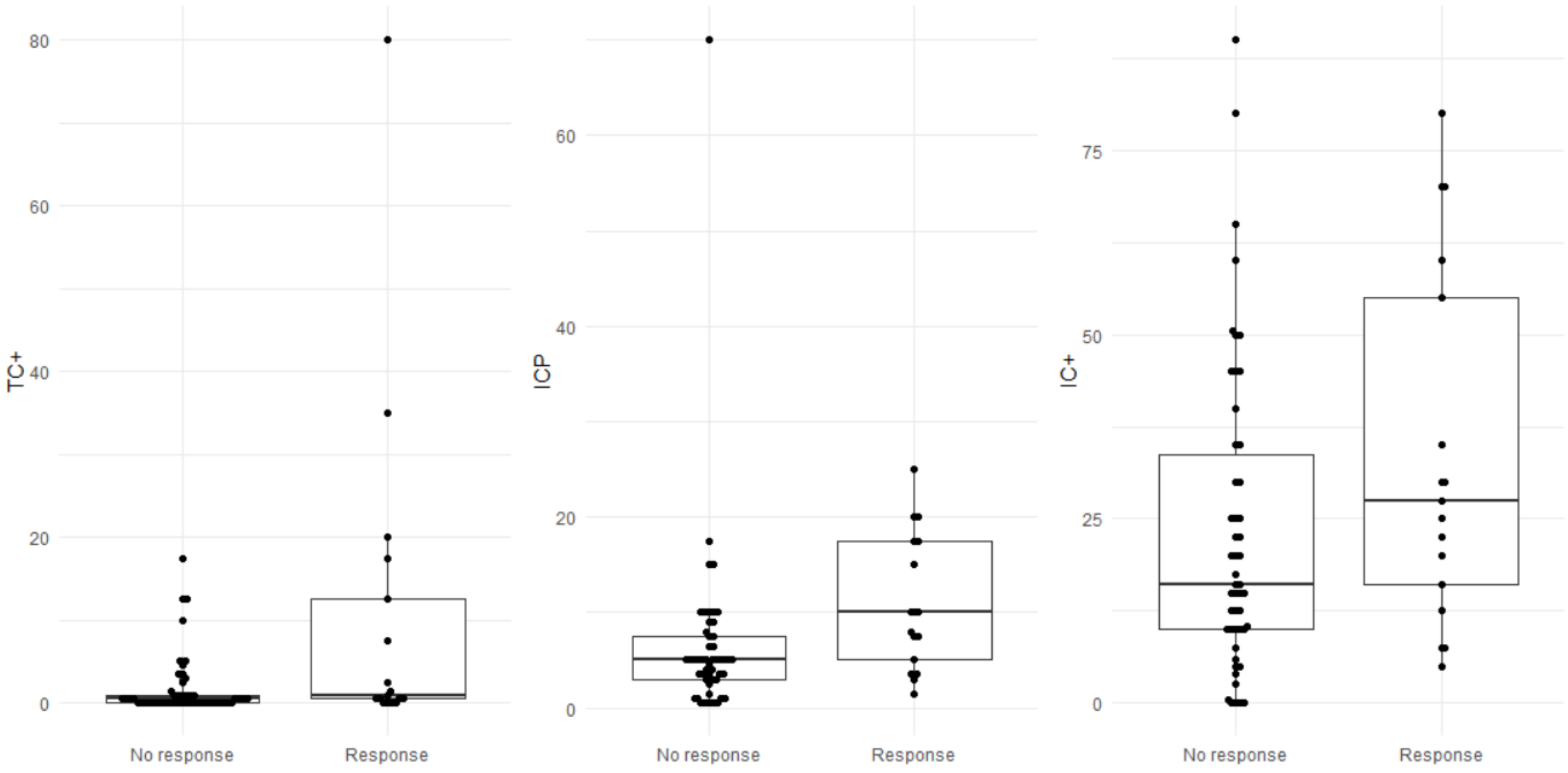

Figure S2: Distribution of the three components scored over responders and non-responders
